# Use of Nonelectrolytes Reveals the Channel Size and Oligomeric Constitution of the *Borrelia burgdorferi* P66 Porin

**DOI:** 10.1371/journal.pone.0078272

**Published:** 2013-11-06

**Authors:** Iván Bárcena-Uribarri, Marcus Thein, Elke Maier, Mari Bonde, Sven Bergström, Roland Benz

**Affiliations:** 1 School of Engineering and Science, Jacobs University Bremen, Bremen, Germany; 2 Rudolf-Virchow-Center, DFG-Research Center for Experimental Biomedicine, University of Würzburg, Würzburg, Germany; 3 Department of Molecular Biology, Umeå University, Umeå, Sweden; University of Kentucky College of Medicine, United States of America

## Abstract

In the Lyme disease spirochete *Borrelia burgdorferi*, the outer membrane protein P66 is capable of pore formation with an atypical high single-channel conductance of 11 nS in 1 M KCl, which suggested that it could have a larger diameter than ‘normal’ Gram-negative bacterial porins. We studied the diameter of the P66 channel by analyzing its single-channel conductance in black lipid bilayers in the presence of different nonelectrolytes with known hydrodynamic radii. We calculated the filling of the channel with these nonelectrolytes and the results suggested that nonelectrolytes (NEs) with hydrodynamic radii of 0.34 nm or smaller pass through the pore, whereas neutral molecules with greater radii only partially filled the channel or were not able to enter it at all. The diameter of the entrance of the P66 channel was determined to be ≤1.9 nm and the channel has a central constriction of about 0.8 nm. The size of the channel appeared to be symmetrical as judged from one-sidedness of addition of NEs. Furthermore, the P66-induced membrane conductance could be blocked by 80–90% by the addition of the nonelectrolytes PEG 400, PEG 600 and maltohexaose to the aqueous phase in the low millimolar range. The analysis of the power density spectra of ion current through P66 after blockage with these NEs revealed no chemical reaction responsible for channel block. Interestingly, the blockage of the single-channel conductance of P66 by these NEs occurred in about eight subconductance states, indicating that the P66 channel could be an oligomer of about eight individual channels. The organization of P66 as a possible octamer was confirmed by Blue Native PAGE and immunoblot analysis, which both demonstrated that P66 forms a complex with a mass of approximately 460 kDa. Two dimension SDS PAGE revealed that P66 is the only polypeptide in the complex.

## Introduction

P66 is an outer membrane protein of Lyme disease and relapsing fever spirochetes [1]. P66 found in the Lyme disease species *Borrelia burgdorferi* is well studied and exhibits dual functions. Firstly, it has been shown to act as an adhesin, which can bind to β3-integrin [2]–[4]; and secondly, it acts as an outer membrane porin [1], [5], [6]. In addition, P66 contains surface-exposed domains [7], [8] and exhibits a certain immunogenic potential [9]. Considering these properties, P66 appears to be an outer membrane protein with promising potential in terms of development of diagnostic tools and prophylaxis for Lyme disease.

The electrophysiological properties of the *B. burgdorferi* P66 protein have previously been studied in detail. P66 is able to form pores in planar lipid bilayers with an unusual high single-channel conductance around 11 nS in 1 M KCl [1]. The channels are nonselective for small anions or cations and exhibit voltage-dependent closure [1], [5]. Although certain other spirochete porins such as those from *Treponema denticola* and *Spirochaeta aurantia* also exhibit extremely high single-channel conductance [10], [11], this is atypical and rare for Gram-negative bacterial porins. To date, besides selectivity and estimated pore diameters, very little is known about the apparent pore size and the structure of these spirochete outer membrane channels.

The channel diameter of P66 has been estimated to be approximately 2.6 nm [5], which is rather large compared to other pore-forming outer membrane proteins [12]. This calculation of the P66 channel diameter was based on the assumption that the conductance of the channel is equal to the conductivity of a simple cylinder of aqueous salt solution. The length of the cylinder was taken to be equal to the thickness of the membrane. This method should be considered as zero-order approximation, because it does not take into account important parameters such as the form of the channel, the field strength inside the channel and repulsion of ions from the hydrophobic zone of the lipid membrane. Therefore, the calculated value of the P66 diameter appears to be somewhat preliminary and its apparent size and structure remained unclear.

To study the size of P66 in more detail, the conductance of the P66 channel reconstituted in planar lipid membranes was studied as a function of the spherical size of nonelectrolytes (NEs) [13]. These polymers were successfully used in the past to determine the effective diameters of a number of polyene-, polypeptide- and protein-channels reconstituted into lipid bilayers [13]–[23]. This method avoids the potentially strong Coulomb interactions that occur when ions were used to probe ion channels containing fixed charges. This study therefore attempted to measure the channel diameter of P66 and to reveal partially its molecular organization in the outer membrane of *Borrelia* species. The results obtained from these experiments were two-fold. First of all, they suggested that the diameter of the P66 channel is much smaller than previously proposed [5]. Furthermore, they are consistent with the view that the active P66 channel is a homooligomer composed of about eight individual channels that may be closed separately by hydrophilic compounds with molecular masses in the range between 400 and 600 Dalton.

## Materials and Methods

### Isolation and Purification of P66 Protein

Pure P66 was obtained by anion exchange chromatography of outer membrane fractions of *B. burgdorferi* B31 [24] as has been described previously [1], [6].

### Planar Lipid Bilayer Assays

The methods used for black lipid bilayer experiments have been described previously [25]. The instrumentation consisted of a Teflon chamber with two compartments containing a 1 M KCl salt solution. The two compartments were separated by a thin wall and connected by a small circular hole with an area of 0.4 mm^2^. The membranes were formed by spreading a 1% (w/v) solution of diphytanoyl phosphatidylcholine (PC) (Avanti Polar Lipids, Alabaster, AL) in *n*-decane over the hole. The porin-containing protein fractions were 1∶1 or 1∶100 diluted in 1% Genapol (Roth) and added to the aqueous phase on one or both sides of the black membrane. The membrane current was measured with a pair of Ag/AgCl electrodes with salt bridges switched in series with a voltage source and a highly sensitive current amplifier (Keithley 427). The temperature was kept at 20°C throughout.

In the experiments carried out to determine the channel diameter of P66, the electrolyte solution contained right from the start 20% (w/v) of an appropriate NE as described previously [20]–[22]. The following NEs were used: ethylene glycol (Sigma), glycerol (Sigma), arabinose (Sigma), sorbitol (Sigma), maltose (Sigma), polyethylene glycol 200 (PEG 200)(Fluka), PEG 300 (Fluka), PEG 400 (Fluka), PEG 600 (Fluka), PEG 1,000 (Fluka), PEG 2,000 (Fluka), PEG 3,000 (Fluka) and PEG 6,000 (Fluka). Polyethylene glycols were the molecules of choice in our studies because in aqueous solutions they have a spherical shape [26], [27]. By statistical analysis of at least 100 reconstituted P66 channels into lipid membranes the single-channel conductance in the presence of the different NEs was evaluated.

The conductivity of each buffer was measured with a multi-range conductivity meter (Knick laboratory conductivity meter 702) using a 4-electrode sensor (Knick ZU 6985 conductivity sensor).

Blocking of P66 conductance by NEs was investigated in the same way as the binding of maltooligosaccharides to carbohydrate-specific porins [28], [29]. The measurements were performed with one single-channel of 11 nS or multi-channel membranes under stationary conditions reached about 90 minutes after adding the protein. At that point NEs were added in defined concentrations to both sides of the membrane while stirring constantly to allow equilibration. Blockage of the channel conductance by NEs could be detected by an impaired ion flux through the channel reducing current and conductance.

The principles of the current noise analysis have been described previously [30]–[33]. Feedback resistors of the current amplifier were between 0.01 and 10 GΩ. The amplified signal was monitored with a strip chart recorder (Rikadenki) and fed simultaneously through a low-pass filter (4 Pole Butterworth Low-pass Filter) into an AD-converting card of an IBM-compatible PC. The digitalized data were analyzed with a home-made fast Fourier-transformation program. The spectra were composed of 400 points and they were averaged 128 or 256 times. The obtained power density spectra were further analyzed using commercial graphics programs.

### Evaluation of the Channel Diameter with Nonelectrolytes

The method of determining the pore size of P66 by using NEs is based on previously published work [17]–[19], [21], [34]. This type of evaluation can be used to determine the size of the channel and its possible constrictions by analyzing the relationship between the single channel conductance in the presence of NEs and their hydrodynamic radii. The determination of the channel radius is based on two principles. First, a salt solution with 20% of a NE will show a reduced conductivity to 40–70%. Second, the decrease in conductivity will only affect the conductance of the channel when the NE used is small enough to penetrate the pore. Otherwise, the solution inside the channel will be free of NEs and the conductance of the channel will be equal to the one observed when using only the salt solution when changes in access resistance and in ion activity can be neglected [16], [18]. Following this, the channel diameter can be considered to be approximately equal to the smallest NE that does not enter the channel and therefore does not reduce its conductance.

To determine a possible constriction inside the channel, the channel filling (*F*) was used in this study in a similar manner as published elsewhere [21]. It is assumed that an ion channel can be treated as an equivalent ohmic resistance (*R*). This assumption can be extended to all channels with a linear current-voltage relationship as has been found for P66 in previous studies [1]. In the limits of experimental errors *R* is assumed to be composed of two parts. One part corresponds approximately to the portion of the channel filled with the NE (*F*) and the other part corresponds approximately to the portion without NE (1–*F*). Using these approximations *R* can be written as [21]:
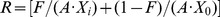
(1)with 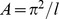. 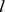 is the channel length and 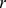 its radius. 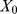 and 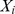 are the conductivities of the aqueous solutions without and with NE, respectively. Assuming that 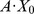 is equal to the ion channel conductance in a solution without NE (corresponding to 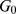), it was shown that the filling (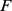) is given by [21]:
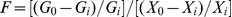
(2)where 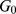 is the single-channel conductance in a solution without NE (1 M KCl), 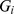 is the single-channel conductance in the presence of a solution containing 20% (w/v) of a NE. Assuming that the filling of the channel by two of the smallest NE (in our study ethylene glycol and glycerol) is close to the maximum possible level, the filling can be calculated in terms of percentage (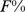) [21]:
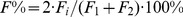
(3)where 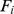 is the filling in the presence of a given NE and 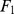 and 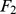 represent filling in the presence of ethylene glycol and glycerol in the bathing solution, respectively.

Analyzing the filling of a channel there exist three different possibilities. If the NEs were considerably smaller than the narrowest part of the channel, it will be filled completely (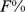 = 100%). When the NEs are close to the size of the constriction of the channel the filling will already be considerably reduced. If the NEs are bigger than the entrance, there will be no NE inside the channel (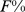 = 0%). Intermediate-sized NEs will fill the channel to an extent inversely related to their sizes (F% between 0 and 100%). These NEs do not fill the channel completely because their size is too big getting stopped somewhere along the channel interior. According to this method, the radius of the constriction zone should be in the range of the radius of the smallest NE that does not pass freely through the channel (filling smaller than 100%).

### Blue Native PAGE and Western Blotting Analyses

Blue native polyacrylamide gel electrophoresis (BN-PAGE) was performed according to previously published protocols [35]. 50 µl (approximately 50 ng) of purified P66 was separated in a 4–13% BN-PAGE. The Native Mark Unstained (Invitrogen) was used as molecular mass standard. For visualization of the proteins, the Blue native gels were silver stained according to a previously published protocol [36].

For Western blotting, a tank blot system (Amersham Biosciences) was used as described elsewhere [37]. Bound antibodies were detected using peroxidase-conjugated anti-rabbit antibodies (DAKO A/S) and enhanced chemiluminescence reagents according to the manufacturer’s instructions (Amersham Biosciences). The production and use of polyclonal rabbit serum against *B. burgdorferi* P66 has been described in previous studies [7], [38].

To reduce the amount of smearing proteins in the BN PAGE the band that showed reaction against the P66 antibody was extracted from a BN PAGE and loaded in a second BN PAGE (BN/BN PAGE) of equal characteristics to the previous one. Extractions from native gels were done using a 0.1% digitonin solution in a shaker over night at 4°C. The pieces of gel containing the P66 band were crushed and mixed with the detergent solution in a ratio 1∶2.

### Second Dimension SDS PAGE and Glycine/Tricine SDS PAGE

The components of the P66 complex were resolved using a 2D well SDS PAGE (NuPAGE Novex 12% Bis-Tris Gel, Invitrogen; Darmstadt, Germany). A vertical strip was excised from a BN/BN PAGE containing the P66 complex. The strip was incubated in three denaturing solutions (reducing, alkylating and quenching solution) during 15 minutes according to the manufacturer instructions (Invitrogen; Darmstadt, Germany).

To improve the resolution and to reach an accurate determination of the molecular weights of the possible protein complex components, the P66 protein band was extracted from the BN/BN PAGE with a 0.1% digitonin solution and loaded in 12% glycine SDS PAGE (self-casted gels) and 16% tricine SDS PAGE (16% Tricine Gel, Invitrogen; Darmstadt, Germany). Proteins were separated under denatured conditions after being boiled for 10 minutes and mixed with SDS sample buffer before loading the gel.

The marker PageRuler Prestained Protein Ladder (Fermentas; St. Leon-Rot, Germany) was used for glycine SDS PAGE while Spectra Multicolor Low Range Protein Ladder (Thermo Scientific; St. Leon-Rot, Germany) was used for tricine SDS PAGE. These gels were stained following a previously published protocol [36].

## Results

### Effects of Nonelectrolytes on P66 Single-channel Conductance

**Figure 1:**
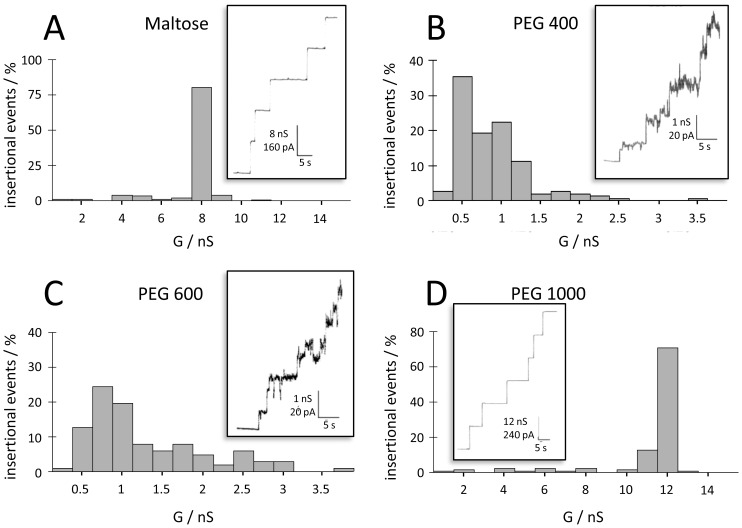
Distribution of the single-channel conductance of P66 in the presence of nonelectrolytes. Histograms were constructed from the evaluation of at least 100 insertional events into a PC membrane in the presence of 20% (w/v) maltose (A), PEG 400 (B), PEG 600 (C) and PEG 1,000 (D) in the bathing solution 1 M KCl. The insets show original recordings of the single-channel current vs. time. The base lines of these recordings represent the zero current level. V_m_ = 20 mV; T = 20°C.

*B. burgdorferi* P66 forms pores with a single-channel conductance of 11 nS in 1 M KCl according to our and other previous studies [1], [5], [6]. The single-channel conductance of *B. burgdorferi* P66 was measured in 1 M KCl solutions containing in addition 20% (w/v) of appropriate NE molecules with defined hydrodynamic radii ranging from 0.26 nm up to 2.50 nm. NEs with a hydrodynamic radius smaller than the pore radius should enter the channel and reduce the channel conductance, whereas larger NEs that cannot enter the channel should have little or no effect on the ionic current [13], [21]. The single-channel conductance in the presence of the different NEs was evaluated by statistical analysis of at least 100 reconstituted P66 channels into neutral PC membranes. Large impermeable NEs with hydrodynamic radii between 0.94 and 2.50 nm (PEG 1,000, PEG 3,000 and PEG 6,000) did not enter the P66 channel and showed no effect on its conductance. However, in the presence of small NEs with hydrodynamic radii up to 0.60 nm, such as ethylene glycol, glycerol, arabinose, sorbitol, maltose, PEG200 and PEG 300, the P66 single-channel conductance decreased proportional to that of the bulk solution conductivity (Table 1). Histograms of four representative NEs measurements together with the recordings of single-channel traces are illustrated in figure 1.

**Table 1:** Average single-channel conductance of P66 in the presence of different nonelectrolytes (NEs) in the bath solution.

Nonelectrolyte	*Mr* (*g/mol*)	*r* (*nm*)	*G ± SD (nS)*	X (*mS cm^-1^*)
None	-	-	11.0 ± 0.48	110.3
Ethylene glycol	62	0.26	6.5 ± 0.23	57.2
Glycerol	92	0.31	5.5 ± 0.27	49.1
Arabinose	150	0.34	7.0 ± 0.31	63.7
Sorbitol	182	0.39	7.5 ± 0.24	57.8
Maltose	360	0.50	8.0 ± 0.34	73.8
PEG 200	200	0.50	6.5 ± 0.37	46.1
PEG 300	300	0.60	7.5 ± 0.27	45.5
PEG 400	400	0.70	0.9 ± 0.28	46.4
PEG 600	600	0.80	0.9 ± 0.47	54.1
PEG 1,000	1,000	0.94	12.0 ± 0.44	49.5
PEG 3,000	3,000	1.44	10.5 ± 0.7	48.9
PEG 6,000	6,000	2.50	10.5 ± 0.51	50.5

Average single-channel conductance *G* and its standard deviation *SD* was calculated from at least 100 conductance steps. The aqueous phase contained 1 M KCl and the corresponding nonelectrolyte at a concentration of 20% (w/v). V_m_  =  20 mV; T  =  20°C. PEG 200 with an equal hydrodynamic radius to maltose was included in this study since maltose displayed some kind of special interaction with the P66 channel. *Mr*  =  molecular mass; *r*  =  hydrodynamic radius; *Mr* and *r* of the nonelectrolytes were taken from previous publications [17]-[22]; X  =  conductivity of the aqueous solutions.

Surprisingly, the presence of PEG 400 and PEG 600 (hydrodynamic radii of 0.70 and 0.80 nm, respectively) in the bathing solution resulted in an exceptional low single-channel conductance of 0.9 nS (less than 10% of the conductance in absence of the NEs) that was not proportional to the bulk aqueous conductivity. This effect appeared to be due to an interaction between the polymer and the channel interior resulting in a conductance block and was further investigated in a separate set of experiments (see below).

### P66 Pore Size Estimation

**Figure 2:**
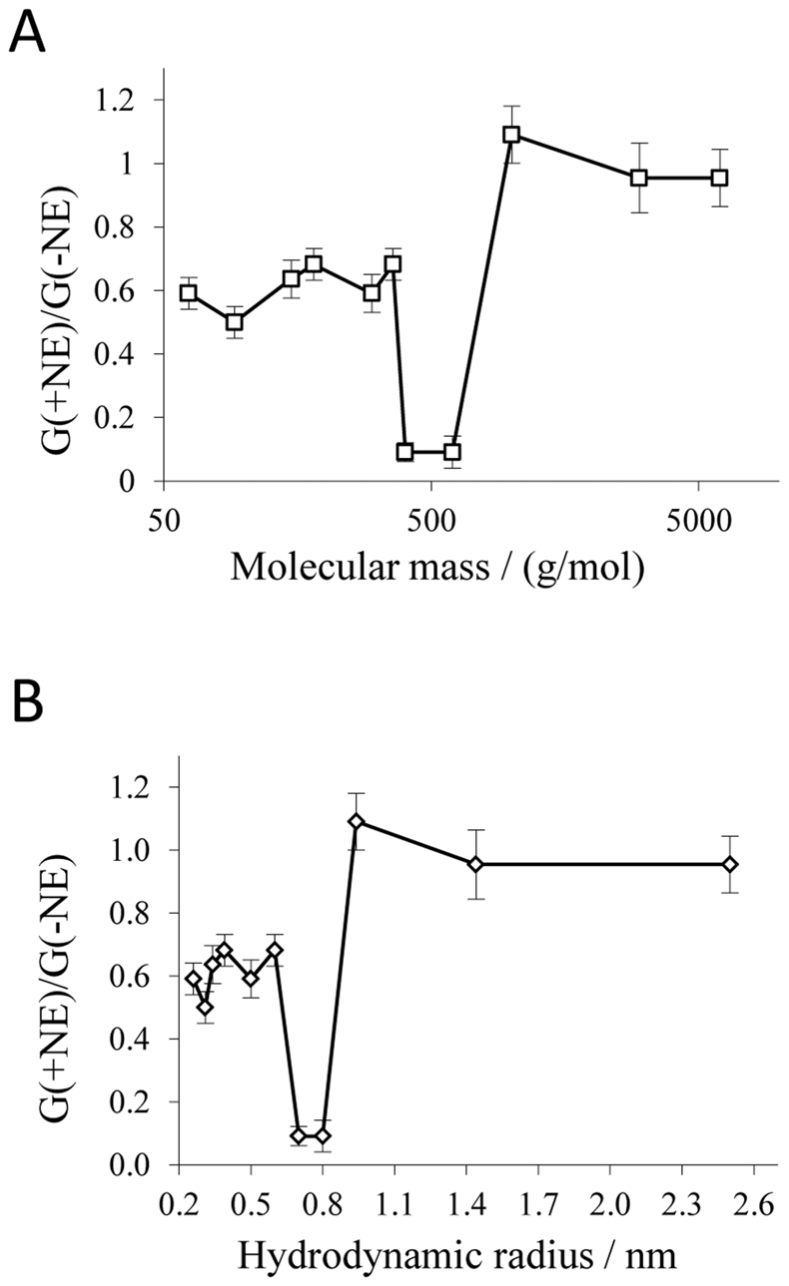
Dependence of the single-channel conductance of P66 on the molecular mass (A) and the hydrodynamic radius (B) of the nonelectrolytes. G_(+NE)_/G_(−NE)_ is the ratio of the mean single-channel channel conductance in the presence of NEs (taken from table 1) to that in the absence of NEs (11.0 nS [1]). Molecular masses and hydrodynamic radii of the nonelectrolytes were taken from table 1. The bars indicate absolute errors.

In order to characterize the pore size, the decrease of P66 conductance was evaluated as a function of the molecular masses and hydrodynamic radii of different NEs (Table 1). The ratios of the single-channel conductance in the presence of NEs to that in the absence of NEs are shown in figure 2. The obtained results suggested that NEs with a mean molecular mass (*Mr*) of ≤600 g/mol and a hydrodynamic radius (*r*) ≤0.8 nm enter the pore whereas NEs with a *Mr* ≥1,000 g/mol and *r* ≥0.94 nm cannot enter the P66 channel. This means that the entrance radius of P66 should be equal to 0.94 nm considering the fact that NEs with such a radius did not decrease its conductance.

**Figure 3:**
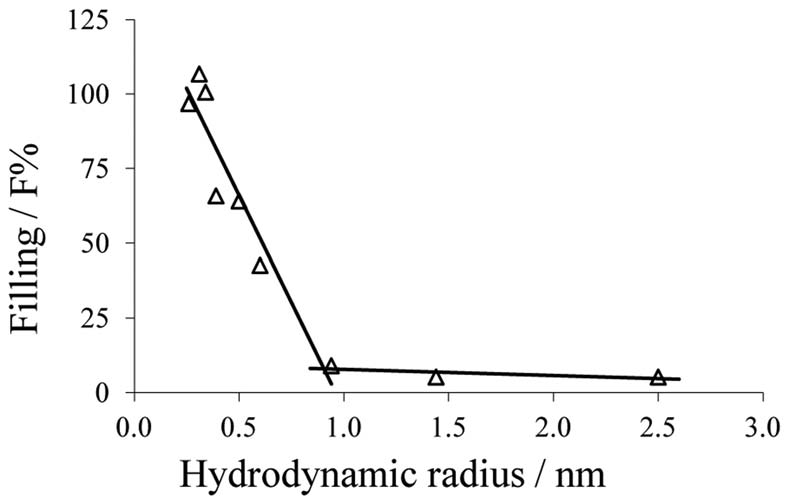
Dependence of the channel filling 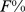 on the hydrodynamic radii of nonelectrolytes. 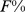
 for each nonelectrolyte was calculated according to Eq. 3. Lines are best fits to the experimental points. The channel filling of maltose, PEG 400 and PEG 600 was not included in this diagram, because the calculated values of 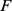 and 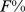 were unreasonably high and could not be used due to possible interactions of these compounds with the channel interior (for details see text). The horizontal lines connect the points derived from measurements in the presence of PEG 1,000, PEG 3,000 and PEG 6,000. The other line regression was used to describe the points for the nonelectrolytes with radii ranging from 0.26 nm to 0.6 nm (ethylenglycol, glycerol, arabinose, sorbitol, PEG200, PEG300). Hydrodynamic radii of the nonelectrolytes were taken from table 2.

It is pertinent to introduce the channel filling in order to correctly determine the size of the P66 channel [21]. The channel filling 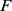 and the channel filling in terms of percentage 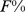 were calculated according to equations 2 and 3 and are listed in table 2. The negative value of 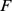 for PEG 1,000 is possible because the channel interior has in the presence of PEG 1,000 a higher conductance than without PEG 1,000 (i.e. 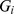 *>
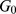
*; see eqn. (2)), which is possible when PEG 1,000 binds water molecules, thereby increasing the salt concentration in the channel [16]. The results of the dependence of *F%* on the hydrodynamic radii of the NEs are shown in figure 3. If the radius of the NEs did not exceed 0.34 nm, 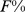 was always close to 100%, as it was the case for ethylene glycol (*r* = 0.26 nm), glycerol (*r* = 0.31 nm) and arabinose (*r* = 0.34 nm). Further increase of *r* caused a decrease in the filling parameter. In this way sorbitol (*r* = 0.39 nm) was able to fill the channel by only 65.8% and PEG 300 (*r* = 0.60 nm) by 42.6%.

**Table 2:** Parameters for the filling of the P66 channel with nonelectrolytes.

Nonelectrolyte	*r* (*nm*)	*F*	*F*%
Ethylene glycol	0.26	0.75	96.8
Glycerol	0.31	0.80	106.7
Arabinose	0.34	0.78	100.6
Sorbitol	0.39	0.51	65.8
Maltose	0.50	0.76	98.1
PEG 200	0.50	0.49	64.1
PEG 300	0.60	0.33	42.6
PEG 400	0.70	nl	nl
PEG 600	0.80	nl	nl
PEG 1,000	0.94	-0.07	9.0
PEG 3,000	1.44	0.04	5.2
PEG 6,000	2.50	0.04	5.2

*F* and *F%* are the absolute ion channel filling and the ion channel filling in terms of percentage, respectively, in the presence of 20% (w/v) nonelectrolytes in the bathing solution 1 M KCl. *F* and *F*% were calculated according to Eq. 2 and Eq. 3, respectively. nl. means neglected: the channel fillings of PEG 400 and PEG 600 were neglected and not included in this table, because the calculated values of *F* and *F*% were without meaning due to possible interactions of these compounds with the channel interior (for details see text). *r*  =  hydrodynamic radii of the nonelectrolyte taken from previous publications [17]-[22].

Channel filling by PEG 400 and PEG 600 was not included in this diagram because 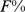 of these NEs exceeded 100% by several orders of magnitude indicating a special interaction between channel interior and NE that was not reported to date in similar studies. Maltose (*r* = 0.50 nm) with a larger radius than sorbitol (*r* = 0.39 nm) filled approximately 100% of the channel, showing also some kind of interaction with the channel interior. Measurements with PEG 200 which has the same hydrodynamic radius as maltose showed an 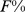 of 64.1%, which was consistent with the inverse decrease of the 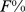 as the hydrodynamic radius increased.

As suggested by the plot in figure 3, the radius of the P66 entrance can be estimated from the intersection point between that segment of 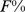, which is dependent on the hydrodynamic radii of NEs and that one forming the lower plateau. The radius of a possible constriction zone should be equal to the radius of the smallest NE that does not pass freely through the channel and do not fill it completely *(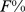
* lower than 100%). In this way, the estimated P66 entrance radius is equal to 0.94±0.1 nm with an inner constriction zone of 0.39±0.1 nm. The error of the estimation of the pore radius may be caused from the standard deviations of the radii of NEs, usually around 0.1–0.2 nm [22].

### Localization of the Constriction within P66 Porin

The method described previously [21] allows also the localization of a possible constriction inside the channel with respect to its position. For this it is a prerequisite that the channels insert always oriented into the lipid bilayer membranes as it was observed for toxins such as Colicin Ia and anthrax protective antigen (PA) [21], [39]. In the case of P66 we used different approaches to study if P66 reconstituted in an oriented manner into the membrane. For these studies P66 was always added to only one side of the membrane, the cis-side. After reconstitution of multiple channels into the membranes, NEs were added exclusively to either the cis-side or the trans-side of the membrane to see if there existed any difference in the NEs access to the channel interior with respect to the addition of the NEs. We could not find any asymmetry with respect to the addition of the NEs, indicating either random insertion of the P66 channels in the membranes or localization of the constriction in the center of the pore. Similar results were also obtained from titration experiment with NEs (see below).

To study the possible orientation of P66 inside the membranes when the protein was only added to one side of the membrane we used also a second approach, which was utilized previously to study asymmetric insertion of the LamB channel into membranes [40]. This approach uses the pH-dependence of the architecture of the loops on the external surface of porins. They have typically long loops looking to the extracellular surroundings while in the periplasmic space they have short turns. A decrease of pH on the side of the external loops led to a collapse of the loop architecture and a closure of the LamB channel and the possibility to study the orientation of the reconstituted channels with respect to their addition to one side of the membrane [40]. Similar experiments were performed with P66 added to only one side of the membrane, the cis-side. When the pH was lowered at both sides to pH 4, the P66-mediated conductance was blocked to approximately 90%. Unfortunately, similar results were obtained when the pH was reduced only at the cis-side or at the trans-side of the membrane (results not shown). Taken together this means again either random orientation of reconstituted P66 in artificial membranes or absence of a remarkable P66 asymmetry.

### Special Interaction of some NEs with the P66 Channel

**Figure 4:**
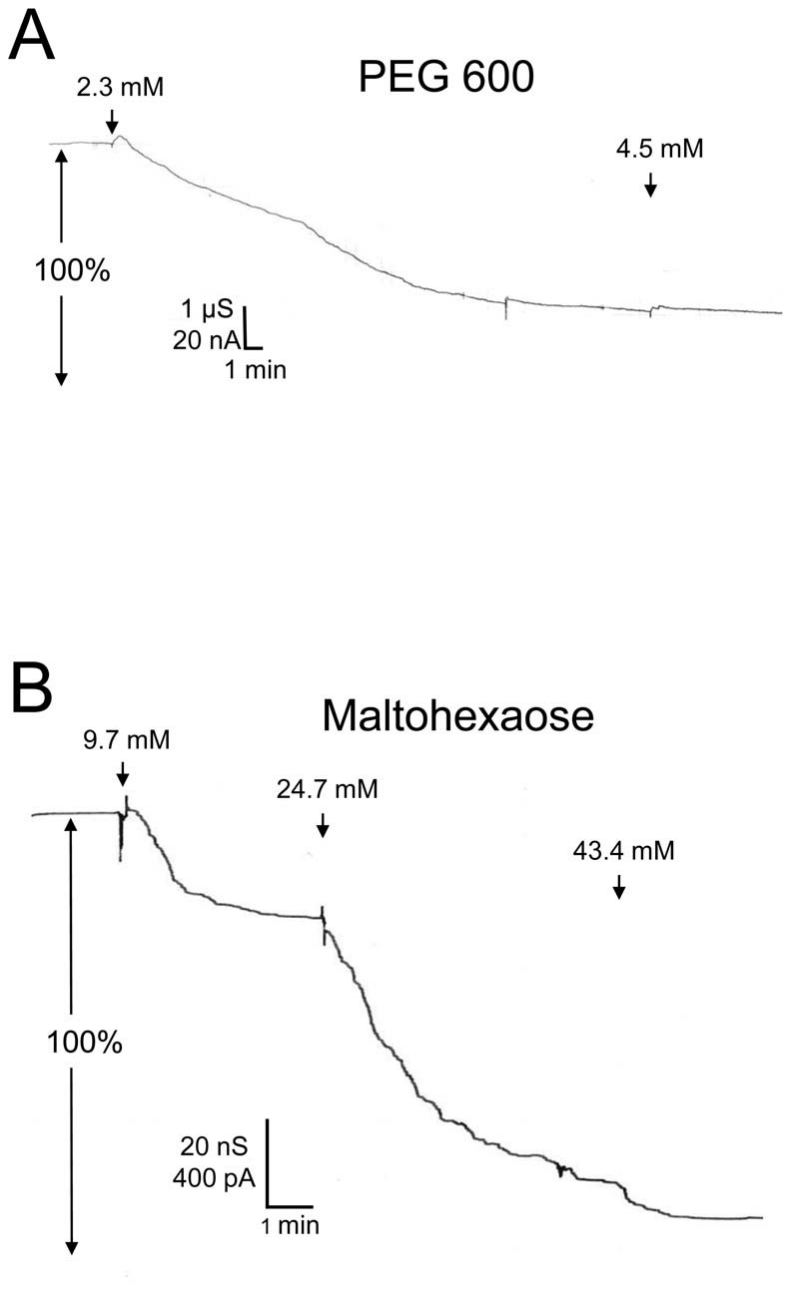
Titration of the P66-induced membrane conductance with PEG 600 (A) and maltohexaose (B). The membrane was formed with PC/*n*-decane. The aqueous phase contained ∼100 ng ml^−1^ P66, 1 M KCl and respective nonelectrolytes in the concentration as indicated; temperature = 20°C; applied voltage = 20 mV.

The results of single-channel measurements with P66 demonstrated that the addition of PEG 400 and PEG 600 caused a dramatic decrease of the channel conductance by about 92% for both PEGs. This is a much larger decrease than that of the bulk conductivity (i.e., by 58% and 51%, respectively) and suggested binding or some other special type of interaction of these PEGs to P66 pores. To investigate the interaction of these PEGs with the P66 channels in more detail, we performed multi-channel titration experiments as described previously for binding of carbohydrates to the LamB-channel [28], [29]. Figure 4 shows two experiments of this type. P66 was added in both experiments in a concentration of about 100 ng/ml to the cis-side of black PC/n-decane membranes. After about 30 minutes the reconstitution rate decreased almost to zero and the membrane conductance became virtually constant. At that time PEG 600 was added in a concentration of 2.3 mM (left side arrow in figure 4A) to the bathing solution on both sides of the artificial lipid membrane. As shown in figure 4A the current decreased slowly after addition of PEG 600. Addition of higher concentrations of PEG 600 (4.5 mM, right side arrow in figure 4A) did not much accelerate this process and did not lead to a considerably further decrease. After about 20 to 30 minutes the residual current was about 20% of the initial one.

Figure 4B shows a similar experiment, where maltohexaose (molecular mass 991 Da) was added to the aqueous phase. Again we observed a substantially slow decrease of membrane conductance after the addition of different concentrations of maltohexaose (arrows in figure 4B). However, it is clear from a critical analysis of the dose-response curve of maltohexaose addition and conductance decrease that it cannot be fitted to a Langmuir adsorption isotherm, indicating some other interaction than simple binding between P66 and maltohexaose. Furthermore, maltohexaose is in principle too big to enter the central constriction of the P66 channel in a more globular form, which also suggests a special interaction between this compound and the P66 channel. In additional experiments, we tested also other NEs such as fructose, glucose, maltose, sucrose and related carbohydrates. Only the addition of small concentrations of PEG 400, PEG 600 and maltohexaose to the bathing solution of P66 containing membranes caused a substantial block of membrane conductance. Other carbohydrates, such as the monosaccharides fructose and glucose or the disaccharides maltose and sucrose did not lead to any block of the P66-induced conductance (data not shown). The P66-induced conductance could be blocked by 80–90% after addition of 4.5 mM PEG 400 or PEG 600 and by approximately 90% after the addition of 45 mM maltohexaose. The kinetics of the decrease of P66-mediated conductance after addition of PEG 400 or PEG 600 was remarkably slow, lasting about 10–30 min, compared to the effect after addition of maltohexaose, which was somewhat faster (see figure 4B). It is noteworthy that even the time course of block of P66-mediated conductance by maltohexaose was considerably slower than that observed previously for the carbohydrate-induced block of the sugar-specific LamB-channels, which correlated well with the time needed to equilibrate maltohexaose in the aqueous phase (at maximum about 5 minutes) [28]–[33], [40]. These results indicated that the interaction between P66 and the NEs PEG 400, PEG 600 and maltohexaose does represent a normal binding process.

Multichannel titration experiments were also performed using artificial membranes with reconstituted P66 channels using NEs (PEG 400 and PEG 600) as substrates with hydrodynamic radii close to the pore exclusion size to study the possible asymmetry of P66. P66 was added in these experiments exclusively to the cis-side of the membranes. The NEs (PEG 400 or PEG 600) were added either only to the cis- or only to the trans-side. The decrease of conductance did not show any difference for any of those NEs depending on one-sidedness of addition, revealing that the interaction of the NEs with P66 showed no asymmetry. This means again either that the NEs could access the constriction site from both sides or that P66 had a random orientation within the membranes (results not shown).

### Effect of PEG 400 and PEG 600 on a Single P66 Channel

**Figure 5:**
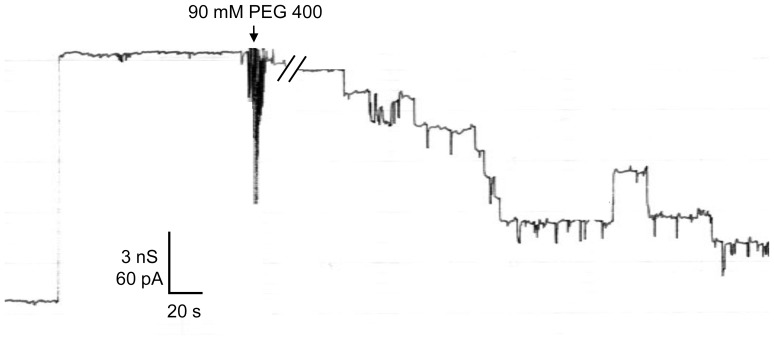
PEG 400-induced blockage of P66 on the single-channel level. Small amounts of highly diluted P66 (1∶1,000 in 1% Genapol) was added to both sides of a diphytanoyl PC membrane. After reconstitution of one single 11 nS P66 unit, 90 mM PEG 400 was added to both sides of the membrane. The P66 conductance was blocked stepwise exhibiting subconductance steps of approximately 1.5 nS; temperature = 20°C; applied voltage = 20 mV.

Additional measurements were performed to study PEG 400, PEG 600 and maltohexaose-induced block of the P66 channel at the single-channel level. An experiment of this type is shown in figure 5. P66 was added in very small concentration to both sides of a black PC membrane. After reconstitution of one single 11 nS P66 unit into the membrane, PEG 400 was added to both sides of the membrane in a final concentration of 90 mM (arrow in figure 5), which allowed a clear observation of the blocking events. The addition of PEG 400 resulted in a step-wise decrease of the current through the channel suggesting a PEG 400-mediated closure of the P66 channel in seven to eight subconductance levels. The conductance of these levels was fairly homogenous and was on average about 1.5±0.2 nS. Only sporadic fluctuations of the subconductance levels were observed indicating that they were not irreversibly closed. They reopened with the same conductance of 1.5 nS in 1 M KCl. We performed also similar experiments with PEG 600 and maltohexaose and received the same results (data not shown).

### Measurements of the Current Noise through the Open and the Nonelectrolyte-induced Closed State of the P66 Channel

**Figure 6:**
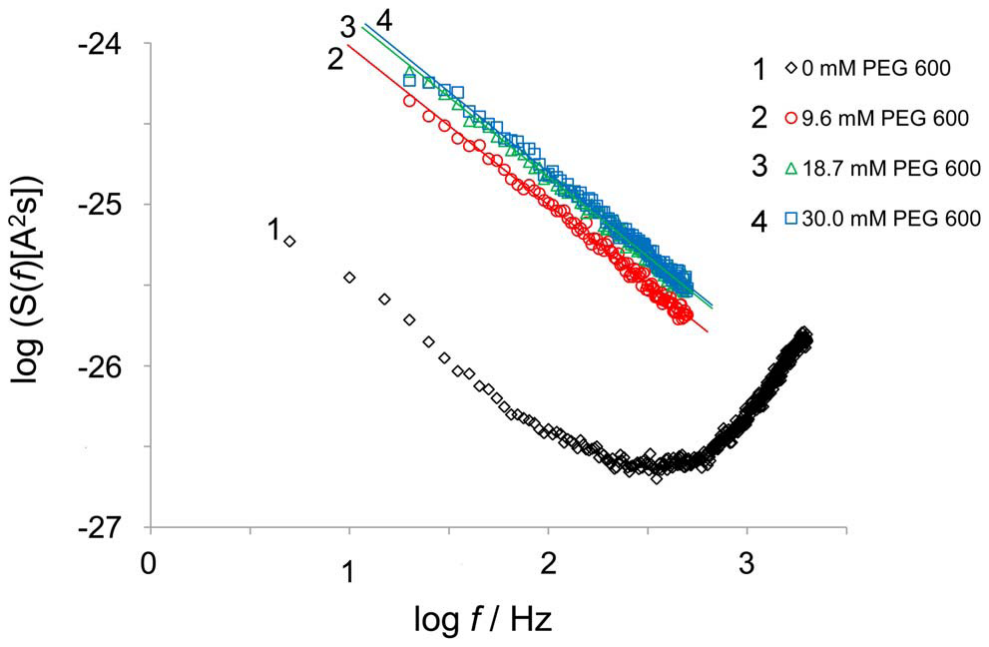
Power density spectrum of PEG 600-induced current noise of 61 P66 channels. Trace 1 shows the control, the aqueous phase contained 1- and maltohexaose-induced current noises resulted in similar power density spectra (data not shown).

The data of figure 4 indicated that the decrease in conductance after addition of PEG 600 is remarkably slow and needed about 20–30 minutes, despite continuous stirring that resulted in much faster equilibration of the NEs within the aqueous phase. To gather some information of the blocking process and its binding kinetics, we studied the current noise of the blocked P66 channels. Parallel to the titration measurements, the frequency-dependence of the spectral density of the current noise was analyzed using fast Fourier transformation. Figure 6 illustrates an example of a measurement with PEG 600. Before addition of NEs, a reference spectrum was taken to obtain the current noise of the open P66 channel, which exhibited 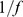-noise in the frequency range between 3 Hz and 100 Hz (figure 6, trace 1). This is typical for open porin channels from Gram-negative bacteria [30]–[33], [41]. The increase of the spectral density at frequencies above about 300 Hz was caused by intrinsic noise of the preamplifier and the membrane capacitance C_m_, which could easily be demonstrated by the measurement of the current noise of dummy circuits containing an appropriate capacitor. The reference spectrum was subtracted from each spectrum taken after the successive addition of NEs in increasing concentrations. Figure 6, trace 2 shows a spectrum taken after addition of 9.6 mM PEG 600 after subtraction of the reference spectrum of trace 1. The spectral density of the current noise increased more than one order of magnitude after the addition of PEG 600. Its power spectrum could also be fitted to a 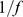-function (see figure 6, trace 2). In further measurements, the concentration of PEG 600 was further increased in defined steps. At the other concentrations of PEG 600 (18.7 mM and 30.0 mM) the power density spectra corresponded to those of traces 3 and 4, respectively, in figure 6, which also could be fitted to 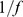-functions. This type of noise is expected for random diffusion processes through open channels [30]–[33], [39]. It has definitely nothing to do with the current noise that is generated by a chemical reaction between a channel and a ligand, such as the carbohydrate-induced channel block of LamB [30]–[33], [40], The spectral density of current noise through P66 channels could also be fitted to 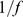 functions after addition of PEG 400, PEG 600 and maltohexaose (data not shown).

### Blue Native (BN-) PAGE Analysis of the P66 Porin Conformation

**Figure 7:**
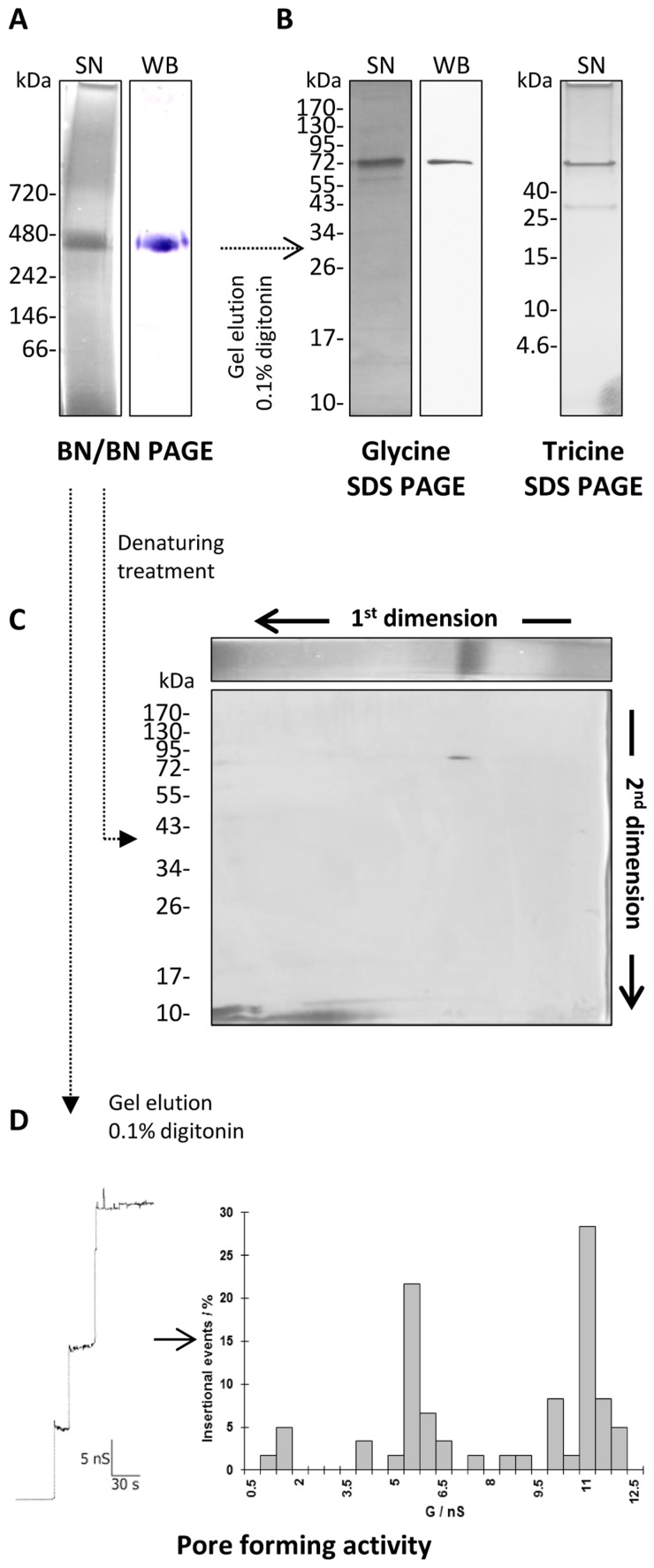
Blue native (BN) PAGE, SDS PAGE and WB analysis of the P66 complex. The complete outer membrane fraction of *B. burgdorferi* B31 was applied to a 4–16% BN PAGE. A 460 kDa band that reacted to the P66 antibody was extracted from the gel and loaded again in a 4–16% BN PAGE (BN/BN PAGE). This gel was stained with silver nitrate (SN) and subjected to a western blot (WB) against P66 (A). The 460 kDa band was extracted from the gel using detergents and resolved again under denaturing conditions in glycine and tricine SDS PAGE (B). A whole lane from the BN/BN PAGE containing the P66 complex was resolved in a second dimension SDS PAGE (C). The elution of the 460 kDa band from the BN/BN PAGE was tested in planar bilayers for pore-forming activity. Step-like increases in the conductance of the membrane were observed after adding the sample to the salt solution (D, left panel) and at least 100 insertional events were summarized in a histogram (D, right panel).

The use of SDS-PAGE to analyze the possible oligomeric structure of P66 did not indicate the formation of any oligomers. SDS is a detergent with denaturing properties for some protein complexes and therefore BN-Page was used, which allows the resolution of oligomeric complexes [35]. When analyzing the prepurified P66 in this native gels a band of approximately 460 KDa appeared (figure 7A, left panel). An immunoblot of the Blue native PAGE was performed using polyclonal antiserum against *B. burgdorferi* P66 [1], [5], [35]. The same band of approximately 460 kDa displayed a strongly positive immunoblot signal (figure 7A, right panel). To optimize the resolution in the high and low molecular range, the P66 protein complex was extracted from the BN PAGE with 0.1% digitonin and subjected to Glycine SDS-PAGE and Tricine SDS-PAGE after adding denaturing sample buffer and boiling (figure 7B). No other protein components were found to be part of the P66 oligomers.

### Two Dimensions SDS-PAGE

Two dimension SDS PAGE was performed to identify possible other protein components within the P66 protein complex. These experiments revealed one single spot of approximately 66 kDa in accordance with the molecular mass of P66. Some smearing of proteins in the BN PAGE could also be observed in the second dimension as faint horizontal lines crossing most of the second dimension of the SDS PAGE. Those bands were not considered to form part of the P66 protein complex.

### Pore Formation by the P66 Oligomers

To check if the high molecular mass band of the BN-PAGE contained any pore-forming activity related to P66 we extracted this protein complex using 0.1% digitonin. The extracted protein was used to determine its pore-forming activity. The sample displayed an 11 nS pore-forming activity in 1 M KCl as shown in figure 7D, left panel. In some cases we observed under the same conditions also a second pore-forming activity of 5.5 nS (see figure 7D, right panel). This activity could be caused by a partial dissociation of the P66 oligomers during their extraction from the BN-PAGE.

## Discussion

### The P66 Pore Diameter is Smaller than Predicted

Estimations based on the previously reported single-channel conductance of 9.6 nS predicted that the P66 channel should have a diameter of 2.6 nm, which is much larger than the diameter of most Gram-negative bacterial porins known to date [5], [12]. The previous rough estimate did not take into account several effects that may influence ion conductance through a channel such as the action of image force, a more hydrophobic interior or that channel friction hinders ion movement [42], [43]. Thus, to get an idea of the effective pore diameter of a channel with such an extremely high single-channel conductance as P66, pore sizing by use of NEs seemed to be a suitable method as has been demonstrated previously [21]. The estimation of the pore diameter by the use of NEs is more precise but still encounters some difficulties and assumptions. For example, the molecular mass of the PEGs is smeared over a considerable range, which could influence the channel filling and thus the estimated radius. However, this effect is accounted and may influence only the standard deviation of the estimated radius. The estimation of the P66 pore size based on our single-channel measurements with different NEs indicated an entrance pore diameter of approximately 1.9 nm with an inner constriction around 0.8 nm. This is definitely much smaller than previously assumed on the basis of pure conductance measurements [5].

A 1,9 nm entrance diameter is within the range of several other Gram-negative bacterial porins and other membrane channels, that were characterized by the use of NEs, such as *Bacillus anthracis* (PA_63_)_7_ (*d* ≈ 2 nm) [44], *Staphylococcus aureus* α-toxin (*d* ≈ 1.35 nm) [22] and the colicin Ia ion channel (*d* ≈ 1 nm) [21]. These three channels exhibit a single-channel conductance of ∼180 pS in 1 M KCl [45], 775 pS in 1 M KCl [46] and ∼90 pS in 1.77 M KCl [21], respectively. P66 has an apparent channel diameter close to the one of *Bacillus anthracis* (PA_63_)_7_, but its single-channel conductance is about 60-fold higher. The high discrepancy between the channel conductance and channel radius cannot only be explained by special effects in the channel lumen. The molecular organization of the P66 complex that is definitely not an artifact of the isolation procedure seems to play a major role as discussed below in more detail.

### The Effects of PEG 400, PEG 600 and Maltohexaose on the P66 Single-channel Conductance are Caused by a Special Interaction with the Channel Interior

Membrane experiments in the presence of 20% PEG 400, PEG 600 or maltohexaose resulted in drastically reduced single-channel conductance. The decrease of ion flux through the channel was significantly greater than the measured decrease of the bulk conductivity after addition of NEs and was also observed during multi-channel measurements which revealed that the P66 conductance could be blocked by 80–90% after the addition of PEG 400, PEG 600 and maltohexaose. Interestingly, kinetics of conductance decrease following addition of these compounds to one or both sides of the membrane was very slow, an observation that differed substantially from titration experiments with substrate-binding porins and channels formed by binding proteins of toxins [29], [39], [45], [47], [48]. This finding enforced the assumption that specific interactions of PEG 400, PEG 600 and maltohexaose with the channel interior resulted in a high partial block of the ion flux through the pore (by about 80 to 90%), which had nothing to do with the expected increase of viscosity of the aqueous phase. We do not have a good explanation for the molecular basis of this block because it was first time observed here. However, measurements of the current noise through open and NEs-induced closed states of P66 channels should enlighten the possible molecular mechanism of this interaction if the binding of the ligands to the channel behaves like a random switch with different on- and off-probabilities (i.e. like by a chemical reaction) [30]. The analysis of the resulting power density spectra of the membrane current as obtained by Fourier-transformation should allow the study of binding kinetics. This has been done previously to study the binding of carbohydrates to the sugar-specific channel LamB and the binding of channel-blockers to channels formed by binding components of different binary toxins [30]–[32], [39], [45]. However, in such a case we would expect that the noise spectrum is of Lorentzian type, i.e. it should be dependent on 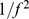, which we definitely did not observe because the noise spectra were dependent on 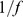 (see figure 6) [30]–[33], [39]–[41], [45], [47], [48].

Open P66 channels exhibited 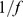-noise before addition of NEs, but also blocked P66 channels showed 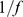-noise with much higher power density after blockage of the P66-induced conductance by NEs. 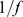-noise is known to describe diffusion processes through open bacterial channels [33], [41]. This means definitely that there was no chemical reaction, e.g. substrate binding, detectable during the interaction of NEs with the P66 channel. This phenomenon is exceptional for binding processes in channels and detailed knowledge to understand blockage of the P66-induced conductance by NEs is missing. However, we have some understanding concerning the generation of 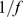-noise by ion current through channels based on a previous study of noise in different open porin channels [33]. In the previous study we could demonstrate that the open channels noise exhibited 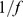-noise for frequencies up to 200 *Hz*. The power density spectrum, i.e. the 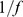-noise was investigated using the empiric Hooge formula [49]:
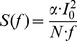
(4)Where 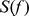 is the amplitude of the power density spectrum at a given frequency 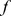, 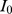 is the current flow through all channels at a given voltage *U*, *N* is the number of open channels in a membrane, and α is the so-called *Hooge*-parameter. The *Hooge*-parameter α was calculated for all membrane channels used in the previous study [33] using the following relationship.
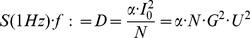
(5)where 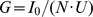
is the conductance of a single channel. In the previous study it has been shown that the Hooge’s parameter α had the form 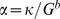 with a slope of b = 1.16 and 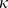 being a new constant factor [33], which means that at a first approximation α is roughly proportional to 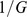. Introducing this relation in equation (5) yields the following relation for D:



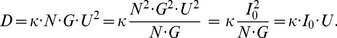
(6)Taken together, an inverse linear relationship has been observed between *α* (i.e. 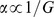) and the single channel conductance, *G*, for the different porin channels, suggesting that the Hooge-parameter decreased for increasing single-channel conductance [33]. Our experimental data (see figure 6) support this view. The addition of PEG 400, PEG 600 and maltohexaose led to a block of the P66 channel by 80 to 90%, whereas the number of channels in the membrane remained the same. This means the single-channel conductance decreased to about 10 to 20% of the initial value. Figure 6 shows that the Hooge parameter *α* increased by a factor of more than 10 caused by channel block, because the noise level of the 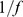-noise of the P66-channels increased at least by a factor of 10. This result suggested indeed that the Hooge parameter *α* is also in the approach used here inversely related to the channel conductance. As pointed out previously we assumed that the passing of an ion through an open channel is - to a certain extent - influenced by non-linear effects between channel wall and passing ion, which increase when the space between channel wall and ions decrease caused by the channel block by the nonelectrolytes [33].

### The Discrepancy between Single-channel Conductance and Effective Diameter Suggested that the Channel-forming Domain of P66 is Composed of Several Subunits

Several experimental observations suggest that the P66 channel is not formed by a P66 monomer alone. First of all, the size of the channel as derived from measurement with NEs does not agree with its extremely high single-channel conductance of about 9–11 nS in 1 M KCl [1],[5]. Furthermore, the stepwise block of the P66 channel with certain NEs occurred in seven to eight substates with a conductance of about 1.5 nS in 1 M KCl. All these results suggested that the P66 channel may be formed by a bundle of pores, which is not a reconstitution or isolation artifact because a similar high single-channel conductance has been found in several laboratories and also for other spirochete porins probably related to P66 [1], [5], [10], [11]. To support this view, purified P66 was investigated by Blue native PAGE (BN-PAGE), a method that allows the determination of native protein masses and oligomeric states of protein complexes [35], [36], [50]. A 460 kDa band observed on the BN-PAGE tends to agree with the oligomeric theory as a P66 octamer would have a molecular mass of 462 kDa. However, since the proteins run in their native conformation, the molecular mass estimation may have an error up to ±15% and a different number of monomers may also possible [50]. It is noteworthy that similar oligomers were observed in a recent study of outer membrane complexes of different *B. burgdorferi* strains [51]. The presence of these complexes was further supported by independent two-dimensional immunoblotting and coimmunoprecipitation assays. Depletion of P66 selectively abolished the corresponding complex [51]. This means that the mass of the oligomeric state of P66 should be in the right order because seven or eight substates with a conductance of about 1.5 nS seem also to match the 11 nS conductance of the oligomer.

Taken together, the results presented here suggested that the individual P66 molecules are forming a high molecular mass protein complex, possibly a heptamer or an octamer. The individual channels in the oligomer act like molecular sieves with a molecular mass cut-off of 182 g/mol and an exclusion size smaller than 1 nm. If that is the case, P66 could be the first known example of a porin constituted from a bundle of eight independent channels in a protein complex. This represents a sharp contrast to the known structure of Gram-negative bacterial porins, which form preferentially trimers [12]. Such a high molecular mass structure was to date only observed in *Borrelia*, but not in any other bacterium or any other living organisms, which means that the structure of the outer membrane channels of this genus of the *Spirochaetaceae* family is similarly different from that of the other Gram-negative bacterial porins as the structure of the borrelial cell envelope lacking LPS.
