# Correction: Use of Nonelectrolytes Reveals the Channel Size and Oligomeric Constitution of the *Borrelia burgdorferi* P66 Porin

**DOI:** 10.1371/journal.pone.0105916

**Published:** 2014-08-11

**Authors:** The PLOS ONE Staff

## Notice of Republication

This article was republished on July 29, 2014, to correct errors in Tables 1 and 2 and Equation 2. Please download this article again to view the correct version. The originally published, uncorrected article and the republished, corrected article are provided here for reference.

## Supporting Information

File S1 Originally published, uncorrected article.
(PDF)

File S2 Republished, corrected article.
(PDF)
